# The impact of revascularization on myocardial blood flow as assessed by positron emission tomography

**DOI:** 10.1007/s00259-019-04278-8

**Published:** 2019-02-26

**Authors:** Robert M. Bober, Richard V. Milani, Ahmet A. Oktay, Fahad Javed, Nichole M. Polin, Daniel P. Morin

**Affiliations:** 1 John Ochsner Heart and Vascular Institute, Department of Cardiovascular Diseases, 1514 Jefferson Highway, New Orleans, LA 70121-2483 USA; 2 Ochsner Clinical School, Queensland University School of Medicine, New Orleans, LA USA

**Keywords:** Coronary artery disease, Coronary flow capacity, Myocardial blood flow, Positron emission tomography

## Abstract

**Purpose:**

Revascularization aims to improve myocardial perfusion. However, changes in regional artery-specific quantitative perfusion after revascularization have not been systematically investigated. It is unclear whether artery-specific thresholds for coronary flow capacity (CFC) and/or relative perfusion predict improved stress perfusion after revascularization. We sought to determine the impact of revascularization based on predefined, artery-specific, severity size thresholds for CFC and/or relative perfusion defects.

**Methods:**

Fifty patients underwent PET imaging before revascularization and then prospectively within 90 days after revascularization. Changes in regional myocardial blood flow (MBF) were stratified based on baseline perfusion abnormalities, baseline reduced CFC, and whether revascularization was performed in that region.

**Results:**

Following angiographic stenosis-directed revascularization, in regions with relative perfusion abnormalities and decreased CFC, stress MBF (sMBF) increased by 0.51 cm^3^/min/g (59%) from baseline (*p* < 0.001). In regions without baseline perfusion abnormalities and yet decreased CFC, sMBF increased by 0.35 cm^3^/min/g (40%) from baseline (*p* < 0.001). In regions without perfusion abnormalities and normal CFC, sMBF did not increase significantly (+0.07 cm^3^/min/g, *p* = 0.56). Patients in whom revascularization was concordant with abnormal PET findings showed increased whole-heart sMBF (+0.22 cm^3^/min/g, *p* < 0.001), but in patients in whom revascularization was targeted only to regions without perfusion abnormalities or low CFC, sMBF did not change significantly (−0.06 cm^3^/min/g, *p* = 0.38).

**Conclusion:**

Revascularization targeted to regions with reduced CFC and relative perfusion abnormalities on baseline PET yielded significant improvements in sMBF. When revascularization was performed in regions without reduced CFC, sMBF did not improve.

**Electronic supplementary material:**

The online version of this article (10.1007/s00259-019-04278-8) contains supplementary material, which is available to authorized users.

## Introduction

In stable coronary artery disease (CAD), revascularization by percutaneous coronary intervention (PCI) or coronary artery bypass grafting (CABG) aims to improve myocardial perfusion for symptom relief or to reduce the risk of myocardial infarction (MI) and death, although randomized trials indicate that the latter goals have not been realized as summarized in the American College of Cardiology guidelines [1]. Despite this aim, regional artery-specific quantitative myocardial perfusion after revascularization in relation to its severity before revascularization has not been systematically investigated. Thresholds for coronary flow capacity (CFC) and myocardial blood flow (MBF) that predict both morbidity and mortality have now been quantified [2, 3]. Accordingly, we tested the hypothesis that the severity of artery-specific quantitative perfusion or relative stress abnormalities could predict stress MBF (sMBF) after revascularization using artery-specific, predefined, severity size thresholds for CFC or relative perfusion defect. We considered that proving or negating this hypothesis might provide an insight into why randomized trials such as [4, 5] and [6] failed to show that revascularization reduces the risk of MI or death in patients with stable CAD by indicating the mechanism by which revascularization improves or does not improve myocardial perfusion.

**Fig. 1:**
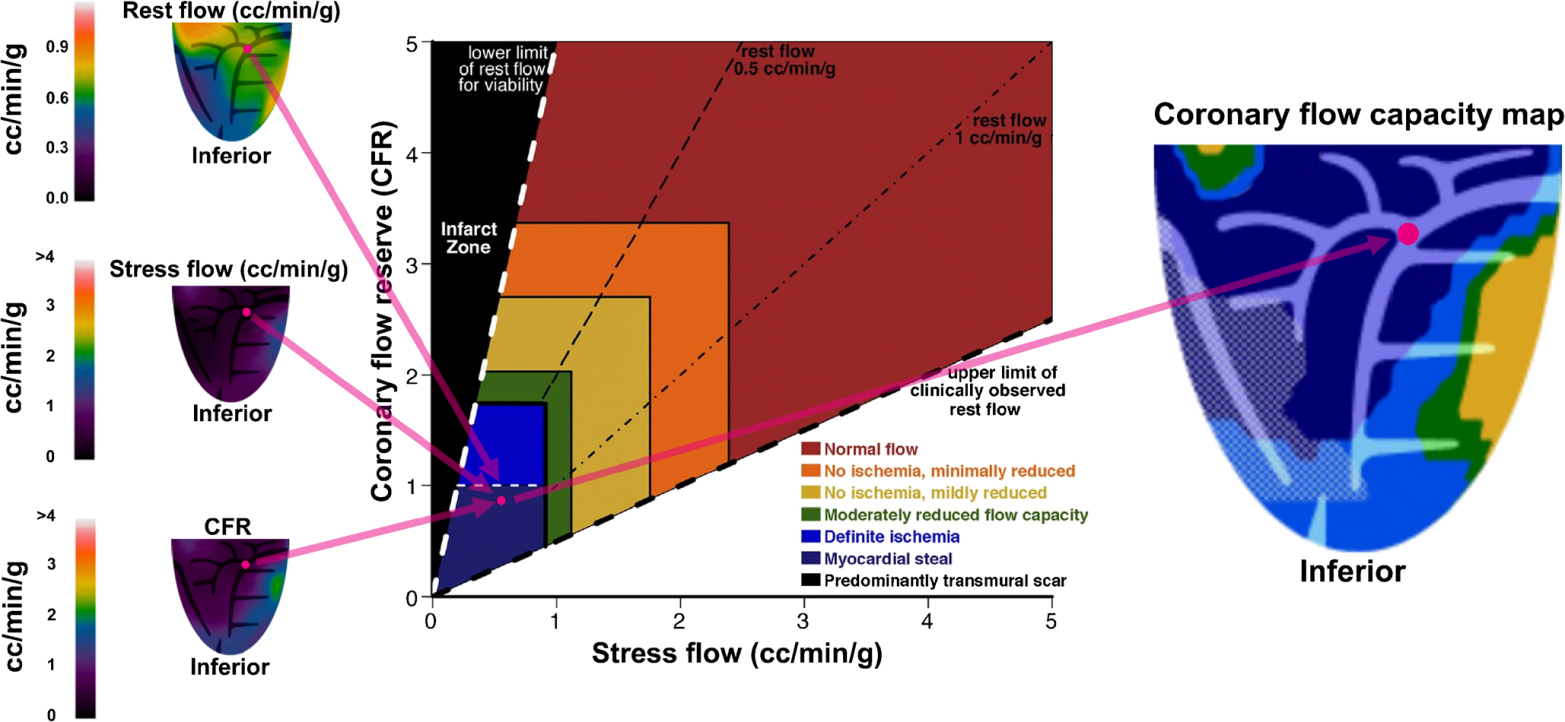
Coronary flow capacity, a framework for determining coronary disease severity, is determined by integrating sMBF, rMBF and CFR. A radial sweep of 21 short-axis slices produces a total of 1,344 pixels. *Left* Each pixel has a value for rMBF, sMBF and CFR and *XYZ* spatial coordinates. One pixel (*pink dots*) is shown to aid understanding. *Center* Each spatially oriented pixel is plotted accordingly and color-coded. *Right* The color-coded pixels are collected to create a flow capacity map. This pixel-by-pixel method allows interpretation of regional and whole-heart MBF and mitigates the impact of resting heterogeneity

CFC is a metric that integrates resting MBF (rMBF), sMBF and coronary flow reserve (CFR) into physiologic severity categories [2]. For assessing coronary perfusion, CFC offers several advantages over traditional metrics such as sMBF and CFR. First, rMBF is regionally heterogeneous, which in turn results in decreased specificity of CFR and sMBF for defining the severity of regional coronary disease [2–9]. Second, CFC has been shown to predict major adverse cardiovascular events and is superior to sMBF and CFR alone [3, 10, 11]. Third, CFC yields a comprehensive platform of physiologic severity that is independent of imaging modality, and thus enables standardization between modalities for describing the impact of CAD and which overcomes the limitations of CFR and pressure-derived fractional flow reserve (FFR) [11]. Finally, CFC simplifies interpretation of the numerous complex datasets of absolute perfusion, CFR and relative images. A detailed explanation of CFC is provided elsewhere [2, 7, 10, 11] and also in Fig. 1.

In a previous retrospective study, we introduced methodologies for assessing the changes in MBF as a result of revascularization [12]. We concluded that revascularization is correlated with improved stress MBF provided only that a stress-induced perfusion abnormality (PA) is present. However, that study was limited by its retrospective design, small sample size, and absence of CFC analysis. The current study was prospective in nature, had a larger sample size with a new cohort of patients, and incorporated CFC into predefined severity size thresholds. We tested the hypothesis that revascularization based largely on angiographic appearance increases sMBF only in locations with reduced CFC and/or a region-specific PA before revascularization, and that in the absence of these prerevascularization features, regional sMBF is not improved by revascularization.

## Materials and methods

The study was approved by the Ochsner Medical Center Institutional Review Board and registered at ClinicalTrials.gov (https://clinicaltrials.gov/ct2/show/NCT02931331). Informed consent was obtained from all individual participants included in the study. Fifty patients underwent clinical PET stress testing (PET1) which triggered revascularization between March 2016 and July 2017. The vessel(s) and method(s) of revascularization were at the treating physicians’ discretion. There was no requirement to base revascularization on the results of PET1. This resulted in unique datasets over a range of size severity PAs, without bias by indication.

Patients were identified consecutively as having PET1-triggered revascularization by an automated daily query of electronic medical records. Once identified, research staff reviewed the records to confirm eligibility and the adequacy of the PET datasets. Patients were contacted and, if they agreed, were enrolled and consented. A non-clinically indicated research PET scan (PET2) was performed within 90 days of revascularization. Thus revascularization was based on “standard practice decisions”, with PET2 measuring its impact on MBF.

**Fig. 2:**
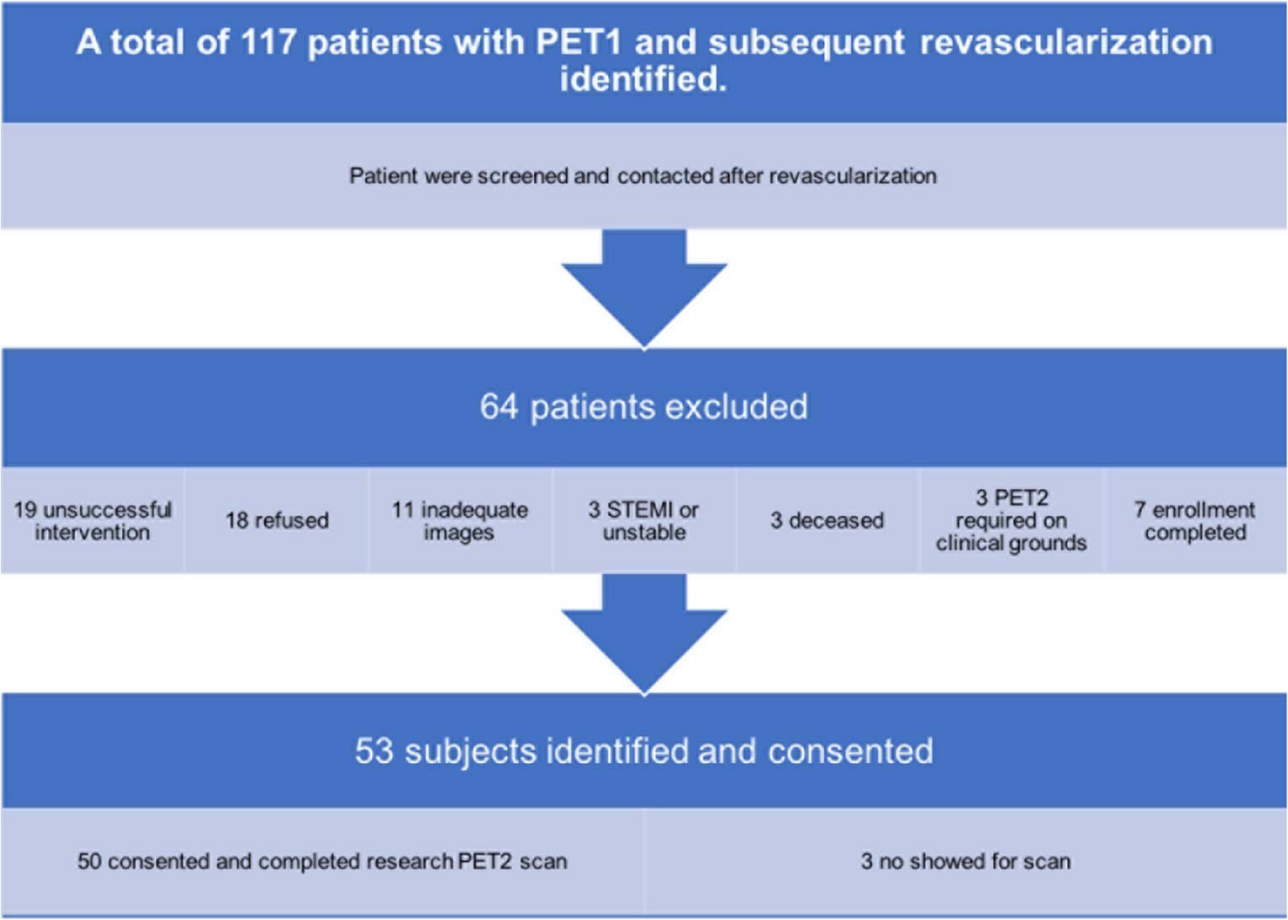
Flow chart of the identification, exclusion and enrollment of eligible patients

Inclusion criteria were age ≥18 years, ability to provide informed consent, and successful PET1-triggered revascularization as described below. Exclusion criteria were ST segment MI (STEMI) within 90 days of PET1, inadequate PET dataset or revascularization images/reports, the necessity for a clinically indicated cardiac stress PET study after revascularization, and revascularization deemed unsuccessful by the operator. A flow chart of the identification, exclusion and enrollment of eligible patients is shown in Fig. 2. While visual estimation of percentage stenosis is notoriously inaccurate, the angiographic percentage diameter stenosis was obtained from the written angiographic report in the patient’s medical record since it was the customary primary driver for revascularization rather than PET perfusion imaging, thereby providing a unique dataset unbiased by indication from the PET data. The “success” of PCI was determined by the operator as poststenosis TIMI 3 flow and less than 20% diameter stenosis. The success of surgical revascularization was determined by the surgeon based on intraoperative findings.

### Image acquisition, reconstruction and quantification of MBF

Patients were instructed to fast for 4 h and to abstain from caffeine and theophylline for 24 h prior to PET imaging. PET imaging was performed using list-mode 2D acquisition on an Attrius PET scanner (Positron, Westmont, IL) with ^68^Ge rod source attenuation correction. Rest and stress emission data were obtained over 7 min beginning immediately upon intravenous injection of 1,295–1,850 MBq of generator-produced ^82^Rb (Bracco Diagnostics, Monroe Township, NJ) at 50 ml/min. Hyperemia was achieved by intravenous infusion of dipyridamole (142 µg/kg/min). Attenuation correction, registration, image reconstruction of topographic views, arterial input selection and computation of MBF were performed as previously reported [12, 13]. For each myocardial quadrant, rMBF, sMBF, CFR and CFC were calculated using FDA-approved software (HeartSee™; University of Texas, Houston, TX) [2].

### Image analysis and definitions of abnormal

Relative PAs can exist in the presence or absence of severe reductions in CFC, and vice versa. Therefore, each left ventricular (LV) myocardial quadrant was categorized in terms of the presence or absence of a significant PA, and separately in terms of the presence or absence of a significant severe reduction in CFC.

**Fig. 3:**
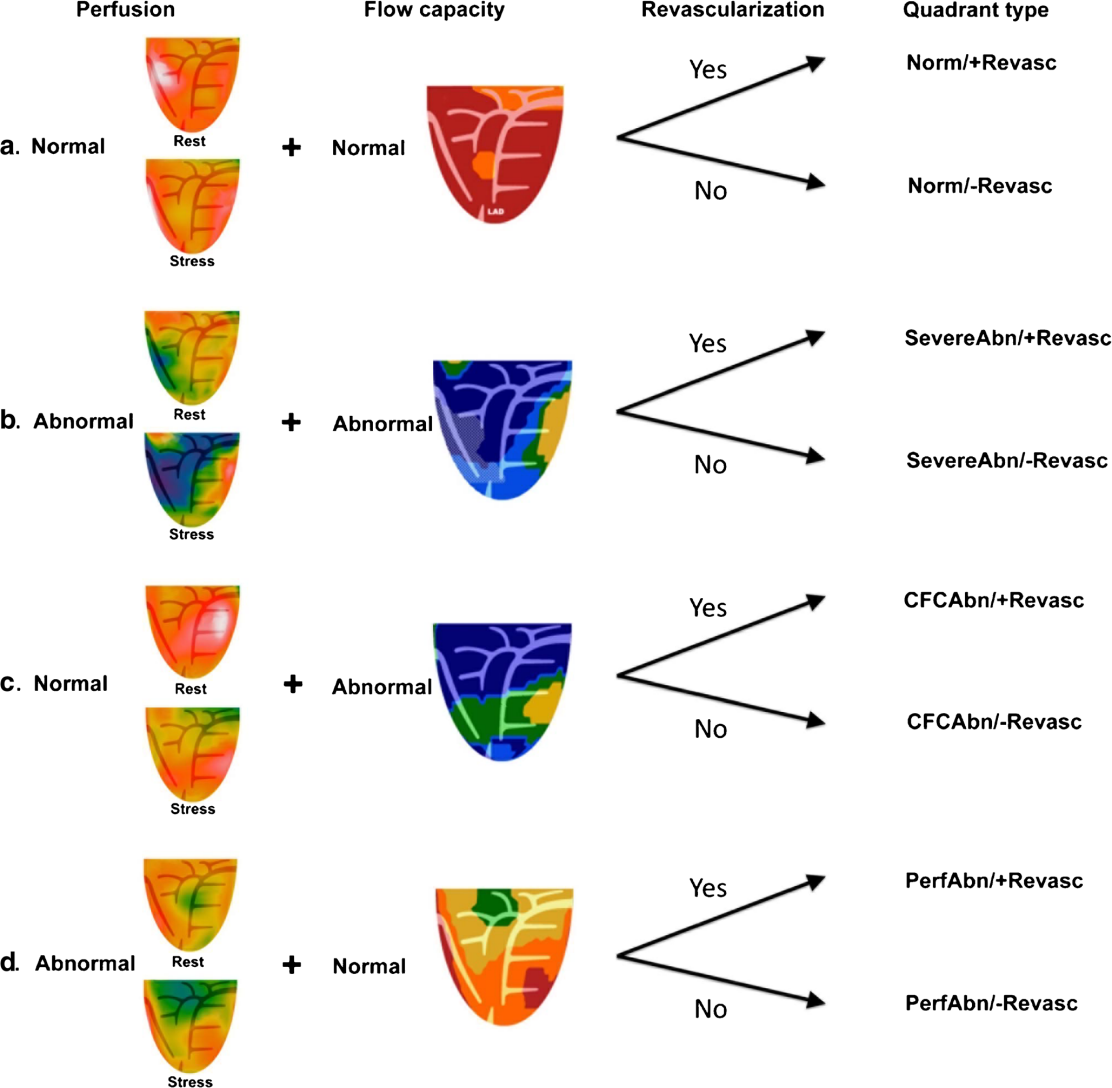
Myocardial quadrant types. Each quadrant’s perfusion and CFC is classified as normal or abnormal. The final categorization is based on whether or not the quadrant received revascularization. See text for details

The size, location and severity of the PA were automatically quantified as the percentage of the topographic maps with relative activity ≤60% of maximal activity. A significant PA was defined as ≥10% change in size and/or severity from the resting scan in a contiguous area of LV myocardium with activity ≤60% of maximum. These regions appeared as green and/or blue defects on relative perfusion images, as shown in Fig. 3. This threshold for declaring significance (size and relative change) identified visually obvious defects and excluded small deviations/variations in radiotracer uptake or reconstruction and stress-induced LV conformational changes [2, 12]. Defects smaller than 10% of the LV myocardium, although possibly not normal, are visually small and could be caused by any of the aforementioned confounders.

A severe reduction in CFC was defined as sMBF ≤0.91 cm^3^/min/g and CFR ≤1.74, on a pixel-by-pixel basis. These thresholds were determined based on clinical features of ischemia [2, 8]. We considered a reduction in CFC significant when a contiguous region spanning ≥10% of the LV myocardium met these conditions. Regions with severe reductions in CFC were color-coded blue on CFC maps, as shown in Fig. 3. These definitions led to four distinct classifications of myocardial “quadrant normalcy”, as shown in Fig. 3:

1. Normal (Norm): <10% PA with <10% severe reduction in CFC
2. Severely abnormal (SevereAbn): ≥10% PA and ≥10% severely reduced CFC
3. Flow capacity abnormal (CFCAbn): <10% PA and ≥10% severely reduced CFC
4. Perfusion abnormal (PerfAbn): ≥10% PA and <10% severely reduced CFC

### Correlation between revascularization and regional myocardial territories

Our methodology for assessing sMBF and CFC responses to revascularization has been described previously [12]. Each epicardial vessel was assigned to a myocardial quadrant (or quadrants) based on its perfusion pattern and review of angiographic data. Appropriate designations of territories were made for left and codominant circulations as needed. Following revascularization, angiographic and/or surgical data were reviewed to determine which quadrants received revascularization. Quadrants were subsequently classified based on their “quadrant normalcy” on the baseline PET scan and whether the quadrant received revascularization. This method led to eight permutations of possible postrevascularization “quadrant types”, as shown in Fig. 3.

### Analysis of revascularization effects

First, we performed a patient-level analysis to determine the overall impact of revascularization on the study population. We subsequently identified three patient categories based on whether revascularization was congruent with the PET findings. “Concordant” patients were defined as those who had revascularization only to territories with a significant PA and/or reduced CFC, “discordant” patients as those who had revascularization only to territories without a PA or reduced CFC, and “mixed-concordance” patients as those who had revascularization to both abnormal and normal regions.

Next, we performed a regional analysis based on the postrevascularization quadrant types. Using this methodology, we intended to determine whether the presence of a PA, or decreased CFC, or both, portended a favorable regional response to revascularization. A representative case example is shown in Supplementary Fig. 1.

### Statistical methods

SPSS version 20.0 (IBM, Armonk, NY) was used for statistical analysis. The results are expressed as means ± standard deviations or as medians and interquartile ranges, as appropriate. Categorical variables were compared using Fisher’s exact test. The normality of the distributions of continuous variables was assessed using histograms, Q-Q plots, and the Shapiro-Wilk test. Because the distributions of the tested continuous variables were largely nonnormal, intrapatient changes in MBF and CFC were assessed using the Wilcoxon signed-ranks test, and quadrant-type changes in MBF and CFC were assessed using the Mann-Whitney *U* test. For all analyses, two-sided *p* values <0.05 were considered statistically significant.

## Results

Fifty patients (70% men, mean age 66 ± 10 years) underwent PET1 and revascularization. The average time between PET1 and revascularization was 34 ± 40 days. Baseline clinical characteristics are shown in Table 1. All patients underwent PET2 within 90 days of revascularization (mean 38 ± 20 days). Medications and physiologic measurements at the time of PET1 and PET2 are shown in Table 2. Of the 50 patients, 41 underwent PCI, 7 underwent CABG, and 2 underwent hybrid revascularization. There were 119 lesions revascularized, treating 111 LV quadrants (56%). The average angiographic visually determined percentage stenosis was 87 ± 11%. FFR was determined in nine lesions. There were 97 stents deployed, and 9 balloon angioplasties and 22 CABG procedures (9 left internal mammary artery, 1 right internal mammary artery, and 12 saphenous vein grafts) were performed.

**Table 1:** Baseline patient characteristics

Characteristic	Value
History of clinical CAD, *n* (%)	40 (80)
Hypertension, *n* (%)	47 (94)
Hyperlipidemia, *n* (%)	46 (92)
Diabetes mellitus, *n* (%)	30 (60)
Prior PCI, *n* (%)	25 (50)
History of CABG, *n* (%)	13 (26)
Left ventricular ejection fraction (%), mean ± SD	43 ± 16
Body mass index (kg/m^2^), mean ± SD	31.9 ± 5.6

**Table 2:** Vital signs and clinical variables at the time of PET1 and PET2

Variable	PET1	PET2	*p* value^a^
Physiologic measurements, mean ± SD			
Heart rate (bpm)	70 ± 12	68 ± 11	0.30
Blood pressure (mmHg)			
Systolic	127 ± 21	130 ± 21	0.38
Diastolic	73 ± 14	76 ± 21	0.38
Rate–pressure product	8,846 ± 2,100	8,832 ± 1,935	0.96
Myocardial blood flow (cm^3^/min/g), median (interquartile range)			
Resting	0.85 (0.65–1.08)	0.88 (0.67–1.07)	0.49
Stress	1.26 (1.03–1.39)	1.35 (1.04–1.7)	**0.002**
Coronary flow reserve	1.38 (1.2–1.7)	1.60 (1.36–1.82)	**0.005**
Abnormality (percentage of myocardium), median (interquartile range)			
Perfusion abnormality	14 (6–21)	2 (0–6)	**<0.001**
Severe coronary flow capacity reduction	33 (13–47)	12 (1–38)	**<0.001**
Tobacco use, *n* (%)	11 (22)	4 (8)	0.09
Medication, *n* (%)			
Beta-blocker	41 (82)	44 (88)	0.58
ACE inhibitor/ARB	41 (82)	41 (82)	1.0
Statin	40 (80)	48 (96)	**0.03**
Calcium channel blocker	18 (36)	20 (40)	0.84
Aspirin	43 (86)	48 (96)	0.16
Platelet Inhibitor	36 (62)	45 (90)	**0.04**
Nitrate	13 (26)	12 (24)	1.0
Diuretic	20 (40)	22 (44)	0.84

*ACE* angiotensin converting enzyme, *ARB* angiotensin receptor blocker

^a^Values <0.05 are indicated in bold type

### Patient-level analysis

**Fig. 4:**
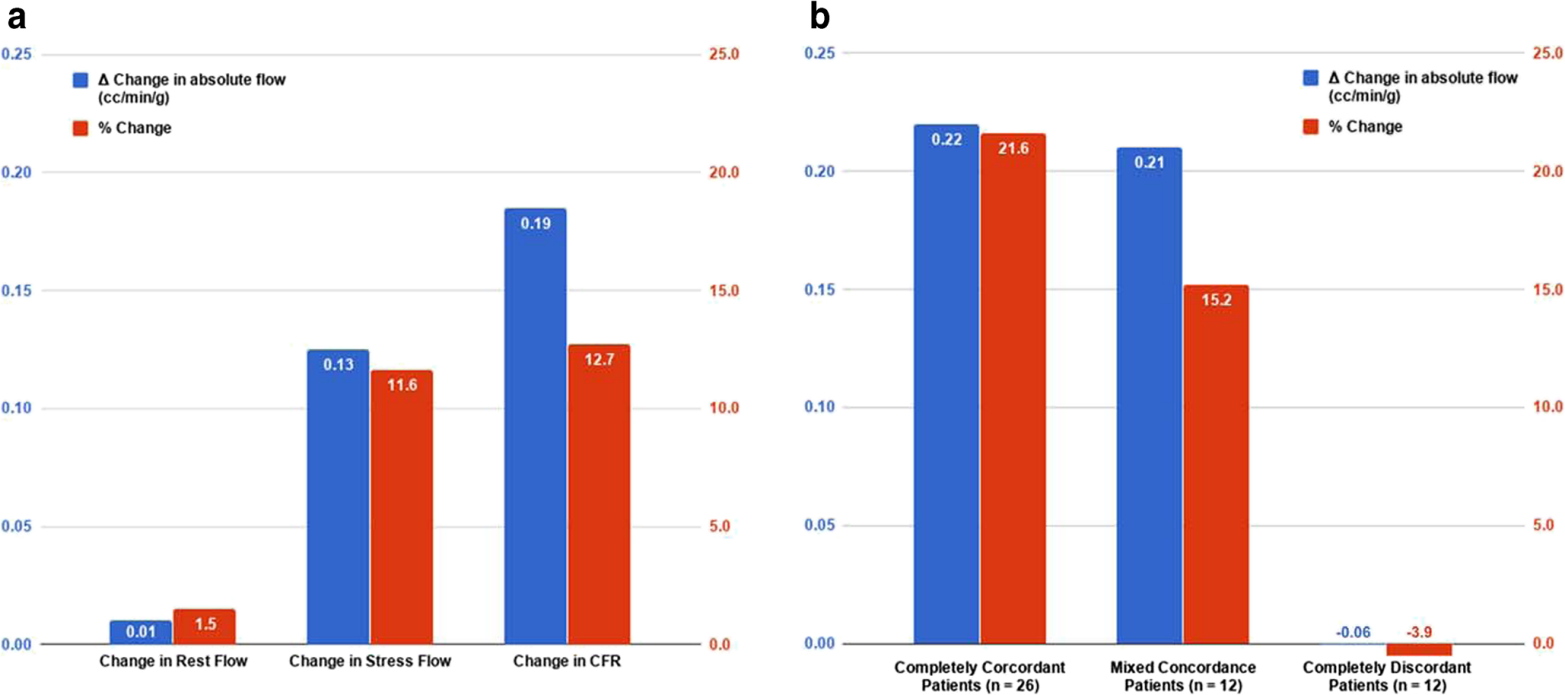
Patient-level analysis of myocardial blood flow following revascularization. **a** Whole-heart changes in rMBF, sMBF and CFR between PET1 and PET2. *Blue bars* represent absolute changes in sMBF; *red bars* represent percentage changes. **b** Whole-heart changes in sMBF based on the concordance between revascularization and perfusion abnormalities. See text for details

The results of the patient-level analysis are summarized in Table 3 and Fig. 4.

**Table 3:** Patient-level analysis

Patient group	Change between PET1 and PET2										
	MBF (cm^3^/min/g)						CFR			Severely reduced CFC (% of myocardium)	
	Resting			Stress			Median (interquartile range)	Percentage change	*p* value^a^	Median (interquartile range)	*p* value^a^
	Median (interquartile range)	Percentage change	*p* value^a^	Median (interquartile range)	Percentage change	*p* value^a^					
All patients (*n* = 50)	0.01 (−0.12 to 0.17)	1.5	0.50	0.13 (−0.04 to 0.41)	11.7	**0.002**	0.19 (−0.06 to 0.41)	12.7	**0.01**	−9 (−28 to 0)	**0.001**
Concordant patients (*n* = 26)	−0.01 (−0.12 to 0.12)	−0.8	0.88	0.22 (0.06 to 0.40)^b^	21.6	**<0.001**	0.31 (−0.02 to 0.46)	23.3	**<0.001**	−24 (−30 to −6)	**<0.001**
Discordant patients (*n* = 12)	−0.08 (−0.19 to 0.05)	−7.0	0.23	−0.06 (−0.33 to 0.08)^b,c^	−3.9	0.380	0.05 (−0.17 to 0.25)	4.1	0.83	0 (−2 to 27)	0.283
Mixed concordance patients (*n* = 12)	0.16 (0.01 to 0.21)	16.5	**0.04**	0.21 (0.06 to 0.51)^c^	15.2	**0.012**	0.16 (−0.12 to 0.37)	11.2	0.38	−18.5 (−36 to −1)	0.060

*MBF* myocardial blood flow, *CFR* coronary flow reserve, *CFC* coronary flow capacity

^a^Values <0.05 are indicated in bold type

^b^*p* = 0.007, concordant vs. discordant patients

^c^*p* = 0.01, mixed vs. discordant patients

### Significant changes in whole-heart MBF and perfusion

Median sMBF significantly increased from 1.26 to 1.35 cm^3^/min/g (*p* = 0.002). Median CFR increased from 1.38 to 1.60 (*p* = 0.005). The median size of PAs decreased from 14.0% to 2.0% (*p* < 0.001). Median percentage of the myocardium with severely reduced CFC decreased from 33% to 12% (*p* < 0.001). Revascularization was concordant in 26 patients. In these patients, sMBF significantly increased from 1.11 to 1.28 cm^3^/min/g (*p* < 0.001), and CFR increased from 1.34 to 1.58 (*p* < 0.001). Revascularization was disconcordant in 12 patients. In these patients, neither sMBF nor CFR changed significantly: 1.43 vs. 1.37 cm^3^/min/g (*p* = 0.37), 1.95 vs. 1.66 cm^3^/min/g (*p* = 0.82), respectively. Revascularization was mixed-concordant in 12 patients. In these patients, sMBF increased from 1.26 to 1.60 cm^3^/min/g (*p* = 0.01).

Among the various concordance groups, there were no significant differences between risk factors and a history of prior revascularization (Supplementary Table 2). Furthermore, risk factors, medication usage at the time of PET1, and PET findings were analyzed in univariable and multivariable analyses (Supplementary Table 3). Among risk factors and medications, none independently predicted improvement in sMBF. A severe baseline PA was a predictor of improvement in sMBF in the multivariable analysis; however, when a severe reduction in CFC was added to the model, a severe baseline PA did not remain a predictor. A severe reduction in CFC was the only independent predictor of an increase in whole-heart sMBF (*β* = 25.338, *p* = 0.032). Furthermore, baseline sMBF, baseline CFR, and baseline percentage of LV with severely reduced CFC were similarly analyzed (Supplementary Tables 4 and 5). In these analyses, a severe reduction in CFC remained the best and sole predictor of (1) any improvement in sMBF and (2) improvement greater than the expected day-to-day variability (>20%) in sMBF (*β* = 0.948, *p* = 0.004 and *β* = 0.015, *p* = 0.004, respectively) [7].

### Regional analysis

**Fig. 5:**
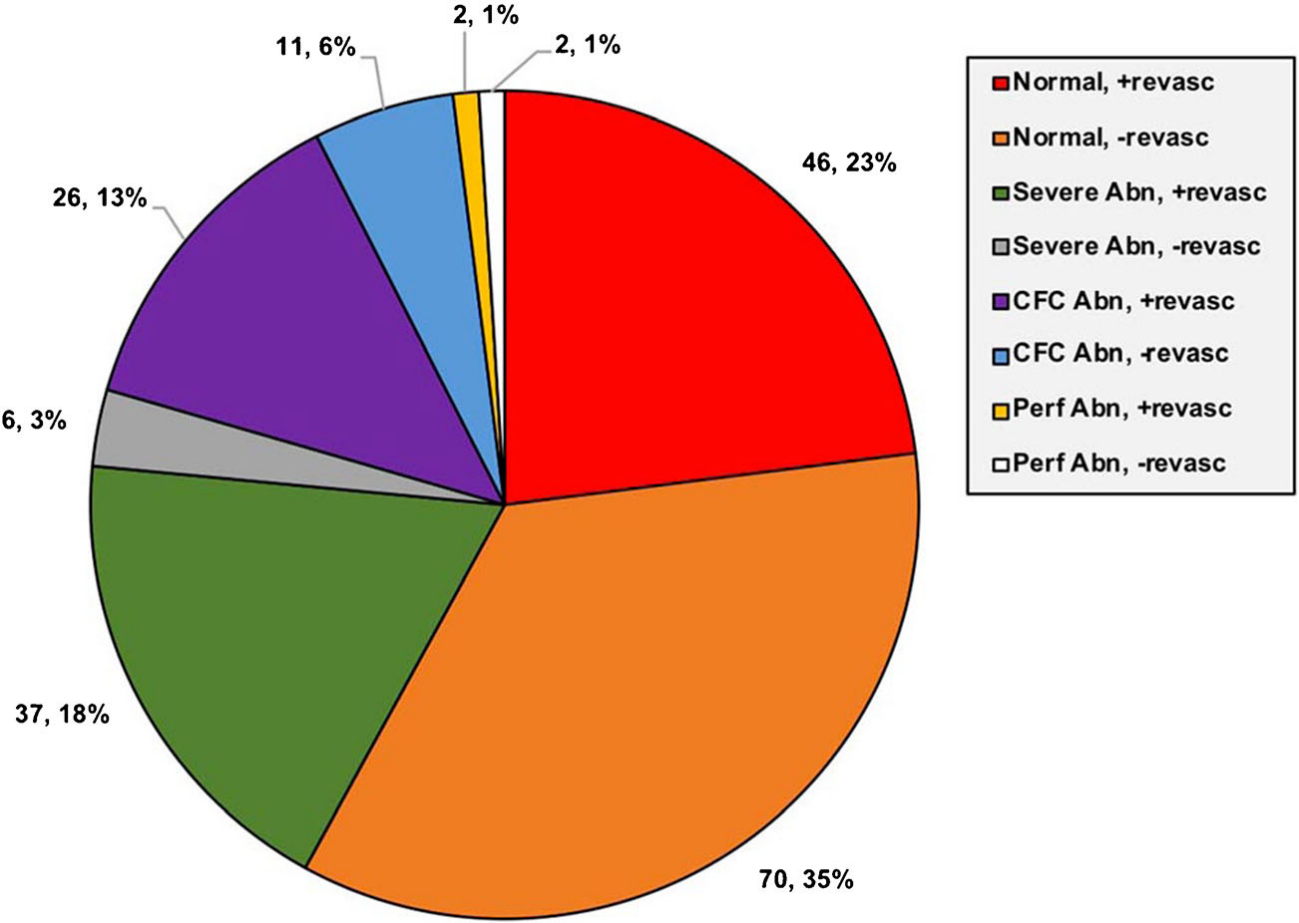
Frequency of quadrant types for all 200 quadrants (50 patients)

**Fig. 6:**
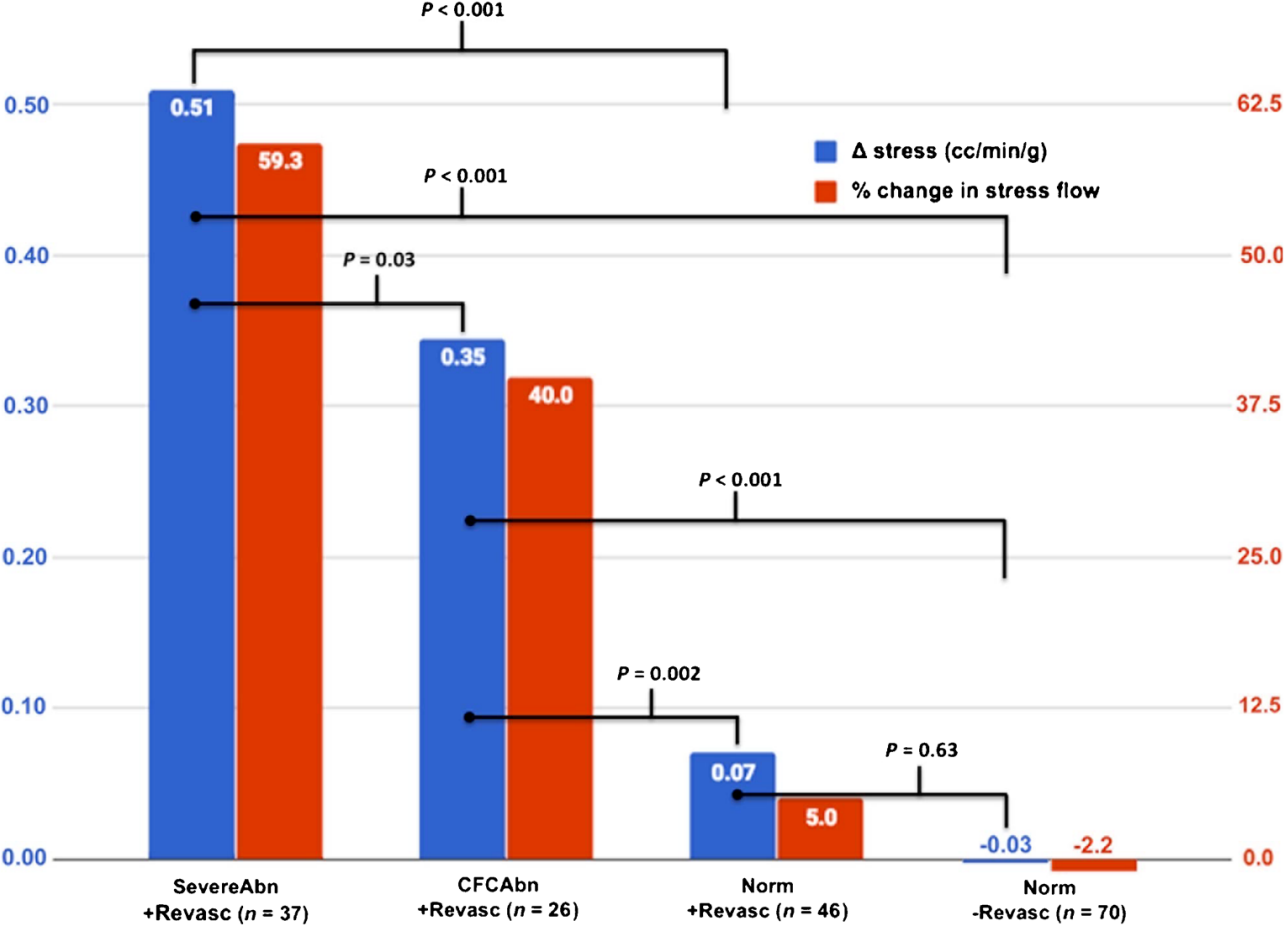
Regional changes in stress myocardial blood flow. *Blue bars* represent absolute changes in sMBF; *red bars* represent percentage changes from baseline

There were 200 regions analyzed (four in each patient: anterior/septal/lateral/inferior). The frequencies of quadrant types are shown in Fig. 5 and Supplementary Fig. 2. The relevant changes in flow, CFR and CFC are discussed below and are summarized in Table 4 and Fig. 6.

**Table 4:** Regional analysis

Quadrant type	Change between PET1 and PET2										
	MBF (cm^3^/min/g)						CFR			Regional CFC (% of myocardium)	
	Resting			Stress			Median (interquartile range)	Percentage change	*p* value^a^	Median (interquartile range)	*p* value^a^
	Median (interquartile range)	Percentage change	*p* value^a^	Median (interquartile range)	Percentage change	*p* value^a^					
Norm/+Revasc (*n* = 46)	0.03 (−0.14 to 0.22)	3.0	0.66	0.07 (−0.31 to 0.29)	5.0	0.56	0.03 (−0.37 to 0.27)	2.0	0.90	0 (−1 to 0.3)	0.49
Norm/−Revasc (*n* = 70)	**−0.08 (−0.18 to 0.02)**	**−8.6**	**0.01**	−0.03 (−0.2 to 0.23)	−2.2	0.77	0.19 (−0.05 to 0.4)	11.4	**0.002**	0.0 (−2 to 0)	0.12
SevereAbn/+Revasc (*n* = 37)	**0.05 (0 to 0.22)**	**11.1**	**0.001**	**0.51 (0.31 to 0.63)**	**59.3**	**<0.001**	**0.52 (0.19 to 0.68)**	**47.3**	**<0.001**	**−12 (−** **19 to −6)**	**<0.001**
SevereAbn/−Revasc (*n* = 6)	−0.03 (−0.14 to 0.1)	−2.1	0.83	−0.03 (−0.18 to 0.07)	−1.4	0.60	−0.01 (−0.22 to 0.07)	−0.5	0.75	1.5 (−4 to 2)	0.92
CFCAbn/+Revasc (*n* = 26)	**0.12(−0.03 to 0.35)**	**18.2**	**0.01**	**0.35 (0.14 to 0.47)**	**40.0**	**<0.001**	**0.11 (−0.11 to 0.36)**	**9.3**	**0.02**	**−9.5 (−15 to −3)**	**<0.001**
CFCAbn/−Revasc (*n* = 11)	0.03 (−0.05 to 14)	7.7	0.45	**0.07 (0.05 to 0.18)**	**9.4**	**0.03**	0.13 (−0.11 to 0.25)	9.9	0.08	−4 (−6 to 2)	0.23
PerfAbn/+Revasc (*n* = 2)	−0.15 (−0.15 to 0.15)	−22	0.50	0.05 (−0.31 to 0.41)	−0.4	1.0	0.27 (−0.05 to 0.58)	27	0.66	0.5 (−5 to 6)	0.66
PerfAbn/−Revasc (*n* = 2)	0.15 (0 to 0.3)	11.1	1.00	−0.09 (−0.4 to 0.22)	−4.4	1.0	−0.28 (−0.53 to −0.02)	−13.8	0.18	−0.5 (−3 to 2)	0.66

*MBF* myocardial blood flow, *CFR* coronary flow reserve, *CFC* coronary flow capacity, *Norm* normal, *SevereAbn* severely abnormal, *CFCAbn* CFC abnormal, *PerfAbn* perfusion abnormal, *Revasc* revascularization

^a^Values <0.05 are indicated in bold type

### Regional changes in rMBF

Following revascularization rMBF in SevereAbn/+Revasc quadrants increased by 11.1% (*n* = 37; +0.05 cm^3^/min/g, *p* = 0.001) and in CFCAbn/+Revasc quadrants increased by 18.2% (*n* = 26; +0.12 cm^3^/min/g, *p* = 0.01). In quadrants that did not receive revascularization (*n* = 89), regardless of relative perfusion or flow capacity abnormality, there was no significant change in rMBF (−0.07 cm^3^/min/g, *p* = 0.30).

### Regional changes in sMBF

Following revascularization sMBF in SevereAbn/+Revasc quadrants increased by 59.3% (*n* = 37; +0.51 cm^3^/min/g, *p* = 0.001) and in CFCAbn/+Revasc quadrants increased by 40.0% (*n* = 26; +0.35 cm^3^/min/g, *p* = 0.01). In contrast, there was no significant change in sMBF in Norm/+Revasc quadrants (*n* = 46; +0.07 cm^3^/min/g, *p* = 0.56) nor in Norm/−Revasc quadrants (*n* = 70; −0.03 cm^3^/min/g, *p* = 0.77). Furthermore, there was no significant difference in the magnitude of the changes in sMBF between these two groups (*p* = 0.63). In quadrants that did not receive revascularization (*n* = 89), regardless of perfusion or CFC abnormality, there was no change in sMBF (median 0.0 cm^3^/min/g, *p* = 0.76).

We made several comparisons between quadrant types. There was a greater relative change in sMBF in SevereAbn/+Revasc quadrants (*n* = 37) than in Norm/+Revasc quadrants (*n* = 46; +59% vs. +5%, *p* < 0.001), indicating that quadrants with a stress-induced PA and reduced CFC showed dramatically better improvement following revascularization than quadrants with neither abnormality. CFCAbn/+Revasc quadrants (*n* = 26) showed a greater change in sMBF than Norm/+Revasc quadrants (*n* = 46; +40% vs. +5%, *p* = 0.002), indicating that improved sMBF could be predicted solely from an abnormal baseline CFC. Finally, there was a significant difference in the percentage improvement in sMBF between SevereAbn/+Revasc quadrants (*n* = 37) and CFCAbn/+Revasc quadrants (*n* = 26; +59% vs. +40%, *p* = 0.03), which demonstrates that the presence of both a PA *and* a low CFC predicts greater improvement in sMBF when compared with a low CFC alone.

### Regional changes in CFC

There were significant decreases in the percentages of the myocardium with severely reduced CFC in the SevereAbn/+Revasc and CFCAbn/+Revasc quadrants: −12% (*p* < 0.001) and −9.5% (*p* < 0.001), respectively. Quadrants of all other types, specifically Norm/+Revasc quadrants, did not demonstrate significant changes.

## Discussion

This study had several important findings. First, revascularization resulted in an average improvement in sMBF, a decrease in the size of PAs, and a reduction in the percentage of the myocardium with severely reduced CFC. Second, patients whose revascularization strategy was congruent with PET-based size severity thresholds showed significant improvement in sMBF. In contrast, those receiving interventions incongruent with PET thresholds showed no improvement in quantitative perfusion metrics. Third, on a regional basis, only those quadrants with reduced baseline CFC demonstrated an increase in sMBF following revascularization. Importantly, and in contrast to the findings of our previous retrospective study [12], even quadrants without a PA on baseline PET demonstrated a postrevascularization improvement in quantitative perfusion metrics when baseline CFC was reduced. In contrast, sMBF in regions without a PA and normal CFC showed no change, regardless of the intervention. The presence of a contiguous area of low CFC was an independent predictor of improvement in sMBF following revascularization, and this prediction was even stronger when a PA was also present in that region. A severe reduction in CFC was the best predictor, adding independent information beyond that of baseline sMBF or CFR. Furthermore, with regard to the magnitude of the change in MBF (or lack thereof) associated with revascularization at various size severity thresholds, our findings are congruent with recently reported invasively derived data obtained before and after revascularization [14].

To our knowledge, this is the first nuclear study to prospectively evaluate whole-heart and regional changes in MBF and CFC resulting from revascularization based on prerevascularization size severity thresholds. Our results provide an insight into the appropriate targeting of revascularization strategies, and also suggest a mechanism that may explain the inconsistency in clinical benefits reported following elective revascularization [15–]. In addition, our investigation revealed that PET imaging corroborates prior invasively derived data [14], while conveniently providing actionable information without procedural risk.

### Revascularization improves MBF and CFC

Appropriately targeted revascularization consistently improved MBF and CFC. Following revascularization, in quadrants with a significant PA and severely reduced CFC rMBF increased by 11%, sMBF increased by 59%, and the percentage of myocardium with severely reduced CFC decreased by 12%. Furthermore, in regions with a large burden of severely reduced CFC but with minimal if any PA, rMBF increased by 18%, sMBF increased by 40%, and the extent of severely reduced CFC decreased by 9.5%. Furthermore, the regional analysis confirmed that improvement in quantitative perfusion metrics was due to revascularization and was not a result of lifestyle changes or medical therapy. Considering recent findings indicating that CFC is directly correlated with mortality and the risk of MI [3], a revascularization strategy focused primarily on improving CFC has the potential to reduce the risk of death and MI.

### Revascularization of quadrants without a significant baseline PA

Based on their angiographic appearance, 72 quadrants without a PA at baseline underwent revascularization. Of these, 46 (64%) had preserved baseline CFC (Norm/+Revasc), and 26 (36%) had significantly reduced baseline CFC (CFCAbn/+Revasc). The differential improvement in sMBF following revascularization between these two quadrant types further demonstrates the value of CFC. While quadrants of these two types had normal relative perfusion images, they contained vessels with visually high-grade disease on angiography, leading to intervention. However, despite visually obstructive CAD, there was no improvement in MBF following revascularization unless CFC was reduced. The differential response in sMBF following revascularization was striking: +40% vs. + 5% (*p* = 0.002) in vessels with and without severely reduced CFC, respectively. Our data highlight physiologic discordances between the widely used functional measurements, relative perfusion imaging (without MBF quantification), angiographic percentage stenosis and CFC, in addition to the impact of revascularization (or lack thereof) on changes in MBF. Furthermore, these discordances were not uncommon: in the current study: about one third of all quadrants were of these two types, and these quadrant types accounted for about two thirds of all interventions. In fact, Norm/+Revasc quadrants were found in 24 of 50 patients (48%) and accounted for 41% of quadrants that received revascularization. Twelve patients had intervention *only* to quadrants with normal relative perfusion and normal CFC. Clearly, patients who underwent revascularization in Norm/+Revasc quadrants were exposed to procedural risk and potential long-term complications without improvement in either sMBF or CFC.

On the surface, a severe reduction in CFC without a corresponding PA seems incongruent. The physiologic explanation for this finding is also the basis for the well-documented and well-described discordance between CFR, sMBF and FFR – that is, diffuse epicardial disease [11, 15]. The appearance of reversibility on radionuclide imaging requires a wide variability in counts throughout the myocardium. The region with the lowest count, when displayed with surrounding regions with high counts, results in what is termed “reversibility.” In the setting of a discrete epicardial stenosis, an abrupt stepdown in radionuclide count density leads to an obvious visual and measurable relative stress defect. However, in the setting of diffuse CAD, there are smaller differentials between areas with higher counts and areas with lower counts. Therefore, reversibility and perfusion defects become less apparent despite concomitant reductions in sMBF and CFC. Furthermore, quantification of diffuse disease on angiography is challenging, subjective and operator-dependent if not measured by intravascular ultrasonography or FFR pullback.

**Fig. 7:**
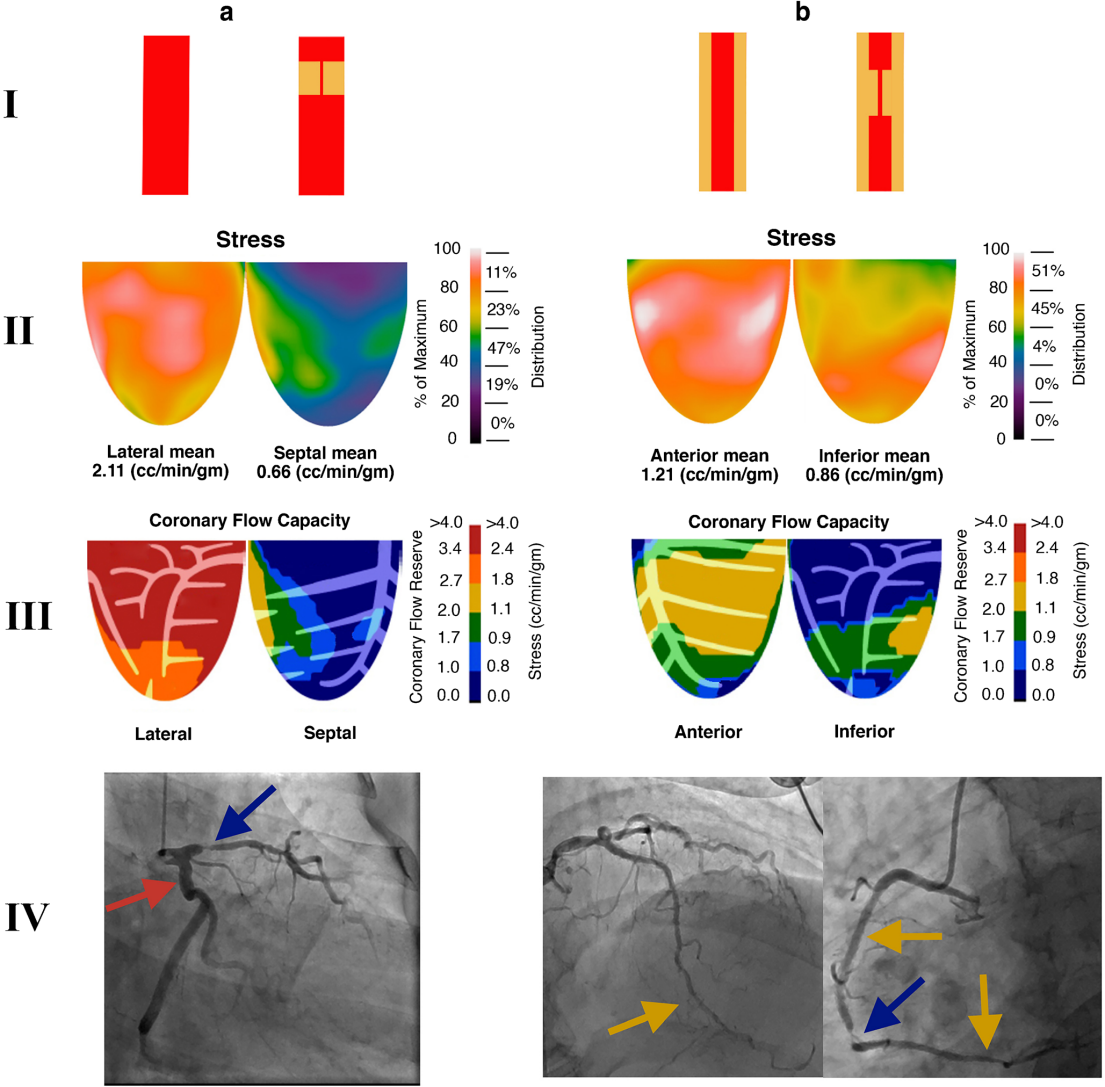
Impact of discrete and diffuse disease on relative perfusion images, stress myocardial blood flow (sMBF) and coronary flow capacity (CFC). For illustrative purposes, only two quadrants are shown. **a***I* Normal epicardial vessel and an epicardial vessel with an isolated high-grade discrete stenosis. The normal vessel perfuses the lateral wall and the vessel with discrete stenosis perfuses the septal wall. *II* sMBF in the lateral wall is near normal (2.11 cm^3^/min/g), whereas the sMBF in the septal wall is markedly reduced (0.66 cm^3^/min/g). This wide variability in sMBF has led to an obvious, large severe relative PA in which septal wall uptake is about 30–40% (0.66/2.11 = 0.31) of that in the lateral wall (*blue-purple* on relative perfusion images).* III* Coronary flow capacity maps show normal CFC in the lateral wall and a severe reduction in CFC in the septal wall. *IV* The angiogram in this patient shows that the left circumflex artery, which is a large dominant vessel, is free of disease (*red arrow*), whereas a visually obvious high-grade stenosis is present in the left anterior descending artery (*blue arrow*). **b***I* Both vessels are abnormal. They both have diffuse epicardial disease, and one vessel also shows a superimposed high-grade discrete disease. *II* The impact of diffuse disease is profound. Stress myocardial blood flow is markedly reduced in the anterior wall (1.21 cm^3^/min/g), which is near the ischemic threshold. The addition of discrete stenosis in the vessel perfusing the inferior wall further reduces sMBF below the ischemic threshold (0.86 cm^3^/min/g). However, the relative drop in perfusion is mild, as the difference in sMBF between these walls is small. This scenario yields a trivial and nonsignificant relative defect (0.86/1.21 = 71% uptake; *yellow zone* in the inferobasilar wall). However, CFC maps demonstrate a moderate reduction in flow capacity at the apex (secondary to diffuse disease leading to a base-to-apex gradient) and a severe reduction in flow capacity in the inferior wall due to the combination of discrete and diffuse disease. *IV* The angiogram in this patient shows that the left anterior descending artery is diffusely diseased and tapers towards the apex (*yellow arrow*). The right coronary artery has several proximal patent stents with mild in-stent diffuse stenosis, and also tapers distally (*yellow arrows*). In addition, a high-grade stenosis is seen in the mid right coronary artery (*blue arrow*)

Diffuse disease was seen angiographically in the vast majority of patients, but was not quantified by any particular metric. However, Gould et al. have reported ranges of flow capacity when coronary disease is present and flow remains above ischemic thresholds. These regions appear yellow and green on flow capacity maps and are indicative of nonobstructive diffuse CAD. In our patient population, 100% of the patients had yellow regions on flow capacity maps. The median percentage of the LV myocardium that was yellow on the flow capacity maps was 36.5% (interquartile range 20.3–46.5%). In other words, all patients had “diffuse disease” and the majority of patients had diffuse disease in vessels perfusing about 20–45% of their LV myocardium. These concepts are illustrated in Fig. 7. This observation could explain why, mechanistically, regions with the combination of a PA and a severe reduction in CFC had a better response to revascularization than regions with just a severe reduction in CFC.

### Revascularization with FFR guidance

FFR-guided revascularization was performed in nine lesions using an FFR threshold of <0.80. Each of these vessels supplied territories without PA and three vessels perfused territories with severe reductions in CFC. All quadrants with normal relative perfusion images and reduced CFC demonstrated improvement in sMBF following FFR-guided revascularization. In contrast, only one of the six quadrants with normal relative perfusion images and preserved CFC demonstrated improvement in sMBF following FFR-guided revascularization. Although the sample size of patients with available FFR data in the current study was small, our results showing discordance between FFR and CFC are consistent with findings reported by others and offer an insight into the impact of revascularization based on these criteria [11, 14].

In a recent study, Driessen et al. [16] enrolled 53 patients without known CAD who underwent serial ^15^O-H_2_O PET before and after FFR-guided revascularization. FFR was routinely measured before and after PCI. sMBF improved by 58% (1.57 ± 0.59 to 2.48 ± 0.91 cm^3^/min/g) after revascularization. In concordance with our data, the percentage increase in sMBF was similar. However, the absolute values of sMBF in the study by Driessen et al. were markedly higher. The reasons for this discrepancy were differences in the patient populations and also possibly in the tracers used. The patient population in the study by Driessen et al. was significantly “healthier” than our population. Exclusion criteria in their study included known CAD, whereas 80% of our population had known CAD, including 26% with prior CABG. Their population was also younger with fewer comorbidities. In addition, Driessen et al. found a baseline sMBF in normal territories of 2.45 ± 0.73 cm^3^/min/g, confirming a relatively healthy patient population with minimal diffuse atherosclerotic disease [2, 9]. Furthermore, plots of the relationships between FFR, sMBF and CFR confirm that the vast majority of lesions with FFR <0.80 had sMBF >0.91 cm^3^/min/g and CFR >1.74 [16]. In other words, CFC was not severe despite a reduction in FFR, a common finding also noted by van de Hoef et al. [11]. Hence, the majority of lesions that received revascularization in the study by Driessen et al. would be of the PerfAbn/+Revasc quadrant type. As noted, in the current study, there were only two quadrants of this type, which is consistent with our population having more advanced CAD.

### Limitations

Although this was a single-center study, we employed validated methods and present the results in a way that provides a generalizable methodology for comparison of quantitative PET data. While quantification of absolute sMBF is commercially available, CFC quantification is currently limited to a few centers. Percentage diameter stenosis was visually estimated by the treating cardiologist, which may have introduced interoperator variability. Symptomatic improvement was not assessed in a blinded and unbiased manner. Due to the study design, objective assessment of symptoms before revascularization was not obtained as enrollment occurred after revascularization. Therefore, conclusions regarding symptomatic improvement with improved MBF cannot be drawn. Furthermore, improvement in global and/or regional LV function could have added more relevance to the findings. However, the sample size of 50 patients in addition to the short duration of follow-up limited such an analysis. Finally, because this study was designed to assess the short-term impact of revascularization, long-term outcome data are not available.

### Conclusion

Revascularization targeted to regions with severely reduced CFC on baseline PET yields improvement in quantitative perfusion metrics particularly if a significant relative PA is also present. Regions without reduced CFC demonstrated no improvement in quantitative perfusion metrics after revascularization.

## Electronic supplementary material

ESM 1 (DOCX 711 kb)

## Abbreviations

CABG: Coronary artery bypass grafting
CAD: Coronary artery disease
CFC: Coronary flow capacity
CFR: Coronary flow reserve
FFR: Fractional flow reserve
MBF: Myocardial blood flow
LV: Left ventricular
MI: Myocardial infarction
PA: Perfusion abnormality
PCI: Percutaneous coronary intervention
PET: Positron emission tomography
rMBF: Resting myocardial blood flow
sMBF: Stress myocardial blood flow
