# Supplementary material for: The impact of revascularization on myocardial blood flow as assessed by positron emission tomography
Source: Eur J Nucl Med Mol Imaging. 2019 Feb 26;46(6):1226–39. doi: 10.1007/s00259-019-04278-8 (PMC6486946; doi:10.1007/s00259-019-04278-8)
Supplement: Supplementary file 1 — (DOCX 711 kb) [file 259_2019_4278_MOESM1_ESM.docx]

Supplementary Material

*The Impact of Revascularization on Myocardial Blood Flow as Assessed by Positron Emission Tomography*

European Journal of Nuclear Medicine and Molecular Imaging

Robert M. Bober, MD^1,2^, Richard V. Milani, MD^1,2^, Ahmet A. Oktay, MD^1^, Fahad Javed, MD^1^, Nichole M. Polin, MD^1,2^, Daniel P. Morin, MD^1,2^

^1^John Ochsner Heart and Vascular Institute, Department of Cardiovascular Diseases, New Orleans, LA

^2^ Ochsner Clinical School, Queensland University School of Medicine, New Orleans, LA

**Address for correspondence:**

Robert M. Bober, MD

[rbober@ochsner.org](mailto:rbober@ochsner.org)

Online Resource Figure 1


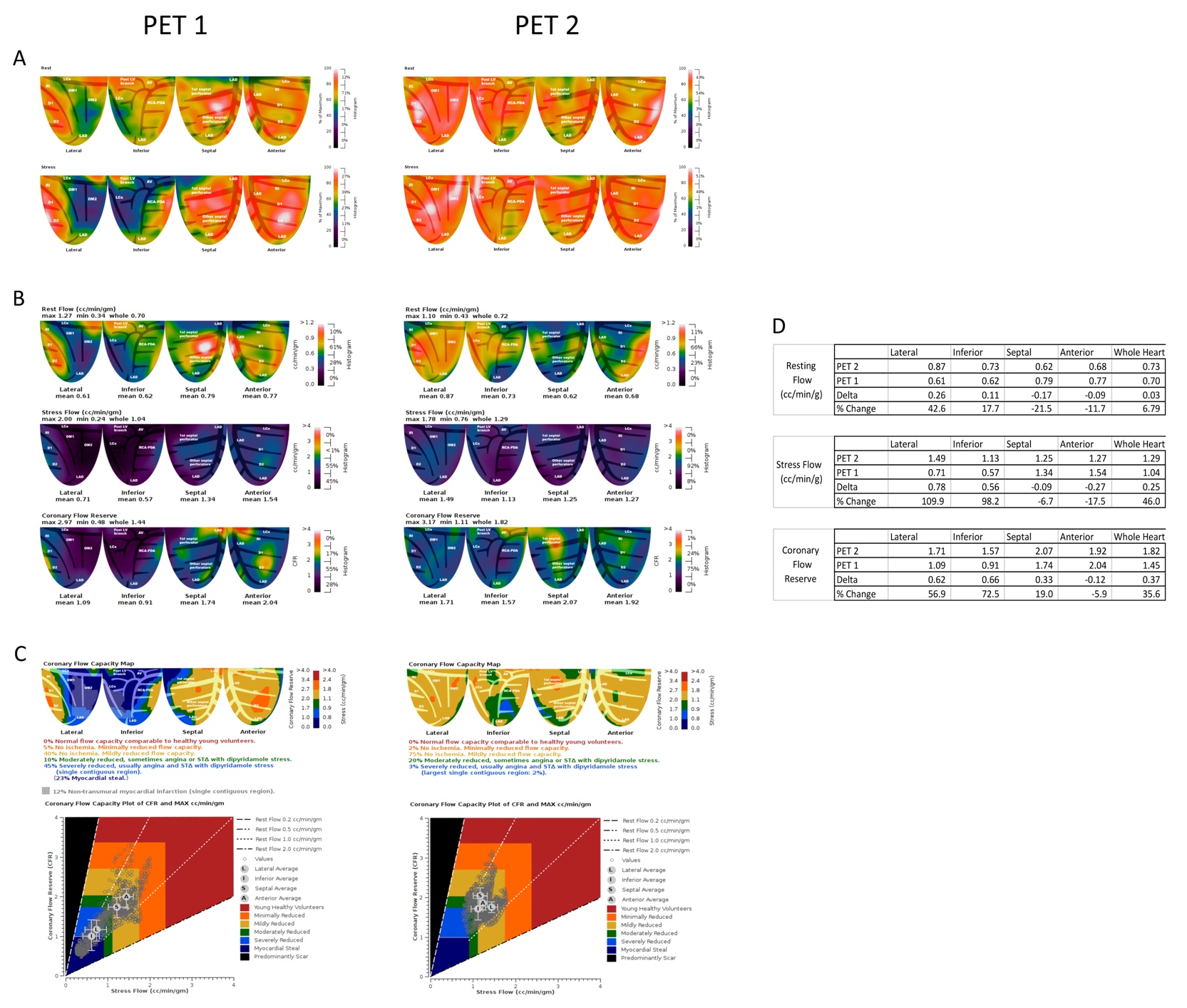


This supplemental figure is a representative case from the study. PET 1 was performed for symptoms of angina and PET 2 was performed 71 days after PCI of the LCX and RCA. (A) Demonstrates a large, severe stress induced relative perfusion defect in the lateral and inferior quadrants comprising 34% of the myocardium. These defects are significant as they represent >10% of the myocardium. There are no significant stress induced perfusion abnormalities in the septal or anterior quadrants. (B) Absolute flow and CFR before and after revascularization with quadrant averages, minimums and maximums. (C) PET 1 coronary flow capacity maps demonstrate severe reduction in CFC in both the lateral and inferior quadrants comprising a total of 45% of the myocardium. As the lateral and inferior walls had significant perfusion abnormalities and were revascularized, they are considered “SevereAbn/+Revasc” whereas the septal and anterior walls are considered “Norm/-Revasc”. After revascularization, severe reduction in flow capacity significantly decreased from 45% to 3% of the myocardium. (D) As a result of revascularization to the lateral and inferior quadrants, rMBF increased 43% and 18% respectively, sMBF increased 110% and 98% respectively and CFR increased 57% and 73% respectively. On a patient level analysis, this patient’s whole heart sMBF increased 46% and rMBF increased 6.8% resulting in an increase in CFR by 36%. On a regional level analysis, this patient contributed 2 “SevereAbn/+Revasc” quadrants and 2 “Norm/-Revasc” quadrants of the total 200 patient quadrants evaluated. This case is an example of “completely concordant” as the patient had revascularization only to territories with perfusion abnormalities and/or reduced flow capacity.

Online Figure 2


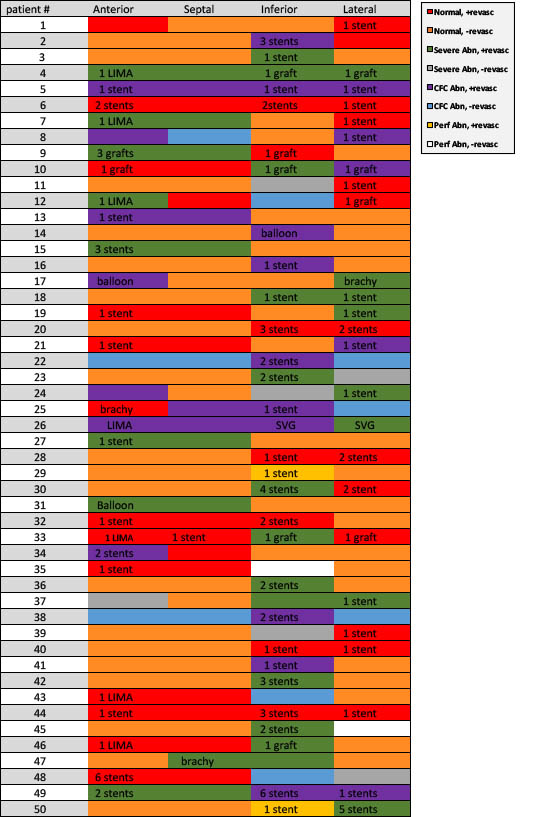


Summary of the quadrant types and revascularization performed in each patient.

Supplemental Table 1

Regional-level analysis comparing PCI vs. CABG

|  | | SevereAbn/+Revasc | CFCAbn/+Revasc | Norm/+Revasc |
| --- | --- | --- | --- | --- |
| PCI | ΔrMBF^*^  [IQR] | 0.05  [-0.01-0.14]  p=0.256  n=24 | 0.10  [-0.05-0.42]  p=0.86  n=22 | -0.07  [-0.17-0.21]  p=0.716  n=34 |
|  | ΔsMBF^*^  [IQR] | 0.52^†^  [0.32-0.78]  **p<0.001**  n=24 | 0.35^‡^  [0.14-0.52]  **P<0.001**  n=22 | 0.05^§^  [-0.31-0.29]  p=0.889  n=34 |
| CABG | ΔrMBF^*^  [IQR] | 0.32  [0.02-0.40]  **p=0.038**  n=13 | 0.14  [0.12-0.23]  P=0.137  n=4 | 0.12  [0.07-0.29]  p=0.256  n=10 |
|  | ΔsMBF^*^  [IQR] | 0.50^†^  [0.31-0.58]  **p=0.004**  n=13 | 0.35^‡^  [0.34-0.43]  P=0.093  n=4 | 0.04^§^  [-0.32-0.18]  p=0.756  n=10 |

* = indicates change between PET1 and PET2 in cc/min/g

^†,‡, §^= ΔsMBF between PCI and CABG regions; p= 0.790, 0.627, 0.644 respectively

Supplemental Table 2

Risk Factors in Patient Concordant Categories

| Risk Factor | Completely Concordant Patients (n=26) | Mixed Concordant  Patients (n=12) | Completely Discordant Patients (n=12) | P value |
| --- | --- | --- | --- | --- |
| CAD | 20 (76.9%) | 9 (75.0%) | 11 (91.7%) | 0.879 |
| Hypertension | 25 (96.2%) | 11 (91.7%) | 11 (91.7%) | 0.992 |
| Hyperlipidemia | 22 (84.6%) | 12 (100%) | 12 (100%) | 0.352 |
| Diabetes Mellitus | 15 (57.7%) | 6 (50.0%) | 9 (75.0%) | 0.816 |
| Tobacco use | 4 (15.4%) | 4 (33.0%) | 3 (25.0%) | 0.828 |
| Prior PCI | 14 (53.8) | 6 (50.0%) | 5 (41.7%) | 0.990 |
| Prior CABG | (9) 34.6% | 1 (8.3%) | 3 (25%) | 0.540 |

CAD, coronary artery disease; PCI, percutaneous coronary intervention; CABG, coronary artery bypass graft

Supplemental Table 3

Regression Analysis Identifying Predictors of Improvement in Whole Heart Stress MBF After Revascularization

|  | Change in Stress MBF | | | | | | | |
| --- | --- | --- | --- | --- | --- | --- | --- | --- |
|  | Univariable Analysis | | Multivariable Analysis 1 | | Multivariable Analysis 2 | | Multivariable Analysis 3 | |
|  | β | P Value | β | P Value | β | P Value | β | P Value |
| Risk factors | | | | | | | | |
| Age | -0.155 | 0.283 |  |  |  |  |  |  |
| Gender | 0.029 | 0.840 |  |  |  |  |  |  |
| Body Mass Index | -0.205 | 0.154* | -1.281 | 0.111 | -1.419 | 0.071 | -1.209 | 0.110 |
| Diabetes Mellitus | -0.318 | 0.024* | -18.191 | 0.053 | -17.277 | 0.058 | -13.707 | 0.121 |
| Hypertension | 0.096 | 0.509 |  |  |  |  |  |  |
| Hyperlipidemia | -0.270 | 0.058* | -23.027 | 0.175 | -23.698 | 0.150 | -16.984 | 0.288 |
| Tobacco use | 0.074 | 0.610 |  |  |  |  |  |  |
| History of PCI | -1.76 | 0.223 |  |  |  |  |  |  |
| History of CABG | -2.47 | 0.083* | -13.434 | 0.193 | -13.843 | 0.167 | -15.817 | 0.102 |
| Medication Use | | | | | | | | |
| Statins | -0.011 | 0.939 |  |  |  |  |  |  |
| Platelet inhibition | -0.092 | 0.525 |  |  |  |  |  |  |
| Beta Blockers | 0.06 | 0.680 |  |  |  |  |  |  |
| Ca channel blockers | -0.039 | 0.788 |  |  |  |  |  |  |
| ACE/ARB | 0.081 | 0.575 |  |  |  |  |  |  |
| PET scan findings | | | | | | | | |
| Severe PA | 0.242 | 0.09* |  |  | 17.187 | 0.050* | 5.445 | 0.579 |
| Severely reduced CFC | 0.443 | 0.001* |  |  |  |  | 25.338 | 0.032* |

PCI, percutaneous coronary intervention; CABG, coronary artery bypass graft; ACE, angiotensin converting enzyme; ARB, angiotensin receptor blocker; PA, perfusion abnormality; CFC, coronary flow capacity

*Significant coefficients for P<0.20 (univariable analysis) or P<0.05 (multivariable analysis).

Supplemental Table 4

Regression Analysis to Determine Which PET Flow Metric Predicts Any Improvement in sMBF After Revascularization

|  | Univariable analysis | | Multivariable Analysis | |
| --- | --- | --- | --- | --- |
| PET Metric | β | P Value | β | P Value |
| Severely reduced CFC | 0.77 | <0.001 | 0.948 | 0.004 |
| Baseline sMBF | -32.66 | 0.01 | 24.15 | 0.221 |
| Baseline CFR | -31.21 | 0.007 | -16.84 | 0.162 |

CFC, coronary flow capacity; sMBF, stress myocardial blood flow; CFR, coronary flow reserve

Supplemental Table 5

Regression Analysis to Determine Which PET Flow Metric Predicts Improvement >20% in sMBF After Revascularization

|  | Univariable analysis | | Multivariable Analysis | |
| --- | --- | --- | --- | --- |
| PET Metric | β | P Value | β | P Value |
| Severely reduced CFC | 0.010 | 0.001 | 0.015 | 0.004 |
| Baseline sMBF | -0.348 | 0.063 | 0.428 | 0.161 |
| Baseline CFR | -0.268 | 0.123 | -0.069 | 0.706 |

CFC, coronary flow capacity; sMBF, stress myocardial blood flow; CFR, coronary flow reserve
